# Prevention of catheter lumen occlusion with rT-PA versus heparin (Pre-CLOT): study protocol of a randomized trial [ISRCTN35253449]

**DOI:** 10.1186/1471-2369-7-8

**Published:** 2006-04-11

**Authors:** Brenda R Hemmelgarn, Louise Moist, Rachel M Pilkey, Charmaine Lok, Marc Dorval, Paul YW Tam, Murray J Berall, Martine LeBlanc, Edwin B Toffelmire, Braden J Manns, Nairne Scott-Douglas, the Canadian Hemodialysis Catheter Working Group

**Affiliations:** 1 Department of Medicine, Division of Nephrology, University of Calgary, Calgary, Alberta, Canada; 2 Department of Community Health Sciences, University of Calgary, Calgary, Alberta, Canada; 3 London Health Sciences Center, University of Western Ontario, London, Ontario, Canada; 4 Department of Medicine, Queen's University, Kingston, Ontario, Canada; 5 University Health Network-Toronto General Hospital, University of Toronto, Toronto, Ontario, Canada; 6 Hopital Regional Geroges L-Dumont, Moncton, New Brunswick, Canada; 7 Scarborough Hospital, Scarborough, Ontario, Canada; 8 Humber River Regional Hospital, Weston, Ontario, Canada; 9 Maisonneuve-Rosemont Hospital, Montreal, Quebec, Canada; 10 Department of Pharmacology and Toxicology, Queen's University, Kingston, Ontario, Canada; 11 Institute of Health Economics, Edmonton, Alberta, Canada

## Abstract

**Background:**

Many patients with end-stage renal disease use a central venous catheter for hemodialysis access. A large majority of these catheters malfunction within one year of insertion, with up to two-thirds due to thrombosis. The optimal solution for locking the catheter between hemodialysis sessions, to decrease the risk of thrombosis and catheter malfunction, is unknown. The Prevention of Catheter Lumen Occlusion with rt-PA versus Heparin (PreCLOT) study will determine if use of weekly rt-PA, compared to regular heparin, as a catheter locking solution, will decrease the risk of catheter malfunction.

**Methods/Design:**

The study population will consist of patients requiring chronic hemodialysis thrice weekly who are dialyzed with a newly inserted permanent dual-lumen central venous catheter. Patients randomized to the treatment arm will receive rt-PA 1 mg per lumen once per week, with heparin 5,000 units per ml as a catheter locking solution for the remaining two sessions. Patients randomized to the control arm will receive heparin 5,000 units per ml as a catheter locking solution after each dialysis session. The study treatment period will be six months, with 340 patients to be recruited from 14 sites across Canada. The primary outcome will be catheter malfunction, based on mean blood flow parameters while on hemodialysis, with a secondary outcome of catheter-related bacteremia. A cost-effectiveness analysis will be undertaken to assess the cost of maintaining a catheter using rt-PA as a locking solution, compared to the use of heparin.

**Discussion:**

Results from this study will determine if use of weekly rt-PA, compared to heparin, will decrease catheter malfunction, as well as assess the cost-effectiveness of these locking solutions.

## Background

In Canada approximately 5 to 40% of hemodialysis patients utilize a central venous catheter for vascular access for hemodialysis [1]. The major complications of these catheters include infection, thrombosis, and poor blood flow resulting in inadequate hemodialysis. Approximately fifty percent of hemodialysis catheters fail within one year of insertion [2,3], with up to two-thirds secondary to thrombosis [4,5].

Inter-dialytic catheter locking with variable concentrations of heparin, usually based on the catheter priming volume [6], is commonly undertaken to prevent thrombosis. The evidence to guide the use of specific locking solutions, including the strength and amount of solution, is limited. Even the National Kidney Foundation Dialysis Outcomes Quality Initiative (K-DOQI) guidelines for vascular access do not provide recommendations for the locking of catheters to prevent thrombosis during the interdialytic period [7].

The critical care medicine and oncology literature have provided some evidence for the use of prophylactic anticoagulant catheter locking solutions to decrease catheter-related thrombosis. While a meta-analysis suggested heparin reduced thrombus formation [8], the heparin dose utilized was extremely variable, ranging from 5,000 units every 12 hours to a continuous infusion of 1 unit/mL (combined with an infusion of total parenteral nutrition). The generalizability of these results to hemodialysis catheters which are larger in size, utilized for longer periods, and require a solution to remain in-situ for up to 72 hours, is questionable.

A few studies have attempted to determine if citrate and heparin have equal efficacy in maintaining hemodialysis catheter patency [9-11], however these studies are limited by their small sample sizes, short follow-up period, and surrogate outcome measures of catheter-related thrombosis prone to measurement error. A recent study comparing citrate 30% and heparin reported no differences in catheter flow problems and thrombosis, however there was a significant reduction in catheter related bacteremia for the citrate arm [12]. Conclusions regarding superiority, or even equivalency, of heparin and citrate for catheter patency can not be made based on the available evidence.

More recently tissue plasminogen activator (rt-PA) has been used in the treatment of catheter-related thrombosis [13-16], and has also been considered as a possible locking solution for the primary prevention of catheter malfunction. Rt-PA preferentially binds to fibrin and thus activates plasminogen in close proximity to the clot, which confines fibrinolysis to the formed thrombus and, in theory, avoids systemic activation. Using a randomized crossover design with 12 patients, Schenk and colleagues [17] demonstrated that use of 2 mg of rt-PA per catheter lumen at the end of each hemodialysis session was superior to the conventional practice of locking each lumen with 2000 units of heparin. After four months, 20% of the heparin locked catheters had a thrombotic event versus 0% in the rt-PA group. While the results of this study are encouraging with respect to rt-PA as a catheter locking solution, the small sample size and single centre enrolment limit the study generalizability and the ability to change clinical practice based on these results. In addition, the cost of using rt-PA after each dialysis session may out-weigh the potential benefit.

The optimal frequency of instillation and dose of rt-PA in the primary prevention of catheter malfunction is unknown. In the study by Schenk et al [17] the dose of rt-PA was 2 mg per lumen (information regarding luminal volume was not provided), instilled after each dialysis session. It may be possible that a smaller dose, such as 1 mg per lumen, or less frequent administration, such as once every week, may result in a decreased incidence of catheter malfunction when compared to the standard instillation of heparin, at a more reasonable cost.

In addition to thrombosis, catheter-related bacteremia is a serious complication of catheter use, with a reported incidence of 2.5 to 6.5 episodes per 1000 catheter-days [18-21]. Several mechanisms are believed to contribute to catheter-related bacteremia, including the formation of an intraluminal thrombosis which may act as a nidus for bacterial biofilm development on the catheter [22,23]. While antibiotic locking solutions have been proposed as a method to treat (.([24], as well as prevent [25], catheter-related bacteremia, their side effects limit their wide-spread use [26]. It is plausible that use of a locking solution which decreases the rate of thrombus formation consequently may also decrease the rate of catheter-related bacteremia [26].

Evidence to date supports the safety profile of rt-PA as a catheter locking solution. Rt-PA is rapidly cleared from the plasma (mediated primarily by the liver), with an initial half life of less than 5 minutes. The dose of rt-PA most extensively studied for treatment of catheter malfunction is 2 mg per lumen, and at these doses systemic fibrinogen levels [27], platelet count, plasminogen level, fibrin degradation products, INR and PTT results all remain unchanged [28]. Two of the largest studies to date of rt-PA use in non-dialysis catheters, which included over 1,000 patients, reported no cases of death, major bleeding episodes, or embolic events attributable to treatment [29,30]. The dose of rt-PA in these studies was 2 mg with a dwell time of up to 2 hours, with a second 2 mg dose administered if function was not restored [29,30]. Thus at low doses rt-PA appears to have relatively few adverse effects.

In summary, the evidence to guide selection of the optimal catheter locking solution in the primary prevention of catheter malfunction is limited. While preliminary data [17] would suggest rt-PA may be an effective locking solution, the optimal dose and frequency of administration are unknown. The Prevention of Catheter Lumen Occlusion with rt-PA versus Heparin (PreCLOT) study will compare the use of rt-PA versus heparin in the primary prevention of hemodialysis catheter malfunction.

## Methods

### Study aims

The primary objective of PreCLOT is to determine if substituting rt-PA (1 mg per lumen) for heparin once per week as a catheter locking solution will decrease the incidence of catheter malfunction, compared to locking with heparin alone (5000 units per mL) after each hemodialysis session. The secondary objective is to determine if substituting rt-PA (1 mg per lumen) for heparin once per week as a locking solution will decrease the incidence of catheter-related bacteremia, compared to locking with heparin alone after each hemodialysis session. An economic evaluation of rt-PA versus heparin in the primary prevention of catheter malfunction will also be conducted.

### Study design and setting

PreCLOT is a randomized controlled trial with blinding of patients, health care providers, and all study staff and outcome assessors. In this study hemodialysis patients with a newly inserted permanent dialysis catheter are randomised to receive rt-PA 1 mg per catheter lumen once per week, with heparin 5,000 units per ml used as a locking solution for the other two dialysis sessions, or heparin 5,000 units per ml (luminal volume) after each dialysis session.

### Ethical considerations

Ethical approval has been obtained in all study sites. Two external bodies, a Data Safety Monitoring Board and a Trial Steering Committee, will monitor study progress.

### Study interventions

The intervention will be to lock the catheter with rt-PA at a dose of 1 mg per lumen, once per week, with unfractionated heparin 5,000 units per ml used as a locking solution for the other two dialysis sessions. Rt-PA will be administered into each lumen initially, with saline added to "top-up" the lock to the full luminal volume. To ensure that rt-PA is administered to the catheter tip, the site of thrombus formation, it will be instilled down each lumen followed by normal saline to a volume adequate to fill the lumen. The control arm will receive unfractionated heparin 5,000 units per ml, with a volume to fill the lumen, administered after each dialysis session. The use of heparin is the current standard of practice in most dialysis centres.

### Identification of eligible patients

Eligible patients are hemodialysis patients with a newly inserted permanent tunnelled catheter, including patients who have had a catheter inserted for the first time (virgin) as well as those who have had either a temporary or permanent catheter at some point in the past (non-virgin). Given the potential for a higher patency rates for virgin versus non-virgin catheters [13], patients will be stratified by virgin vs non-virgin status. Inclusion and exclusion criteria are outlined in Table 1.

**Table 1:** Study inclusion and exclusion criteria

***Inclusion criteria:***
• End stage renal disease patients with newly inserted permanent, tunnelled, dual-lumen catheter
• Naïve to study but not naïve to catheters (both virgin and non-virgin catheters will be included)
• Expected to use catheter, and to dialyze at study centre, for at least six months
• Frequency of hemodialysis 3 times per week
• If indication for catheter was replacement for catheter related infection patients will be eligible after the infection has been treated and the patient has been off antibiotics for 3 hemodialysis sessions
• Patient or legal representative able to provide written consent
• Eighteen years of age or older
• Baseline INR ≤ 1.3
• Baseline platelet count ≥ 60 × 10^9^/L
***Exclusion criteria:***

• Use of systemic anticoagulation (if indication for anticoagulation is catheter patency patients may be eligible if the systemic anticoagulation is discontinued and baseline INR is = 1.3)
• Insertion of a new permanent catheter by a guide-wire exchange procedure
• Insertion of a new permanent catheter into the femoral vein
• Current use of antibiotics for catheter-related bacteremia (see inclusion criteria above)
• Major hemorrhage in the prior 4 weeks, defined as bleeding resulting in a drop in hemoglobin of greater than 20 g/L or bleeding requiring transfusion of packed red blood cells with other clinical evidence or suspicion of bleeding
• History of intra-cranial bleed in the prior 4 weeks
• Intra-cranial or intra-spinal neoplasm (current)
• Allergy or intolerance to rt-PA or heparin or its constituents
• Active pericarditis – defined by the presence of a pericardial rub
• Weight ≤ 30 kg
• Patient pregnant or lactating
• Child bearing potential (i.e. pre-menopausal woman who is not using a reliable method of contraception)
• Major surgery in past 48 hours (CABG, organ biopsy, puncture of non-compressible vessels), or scheduled for major surgery during the study period
• Involvement in another randomized drug trial
• Presence of a fever as defined by a temperature > 38.2°C

Patient recruitment will occur over a two week period following catheter insertion, during which time centre specific catheter management will take place. The patient will be eligible for randomization after the fourth hemodialysis session, if sessions three and four were successful (defined as a mean blood flow of ≥ 300 ml/min). The two week eligibility period is included for two reasons: 1) the risk of local bleeding following catheter insertion may be slightly increased using a locking solution of heparin 5,000 units per ml, therefore catheter management during the initial two week period will be at the discretion of the study centre; and 2) catheter malfunction which occurs within two weeks of insertion is more likely due to a mechanical cause rather than thrombosis.

### Randomization and study blinding

Following informed consent, patients will be randomized in blocks of four, and stratified by study centre and virgin versus non-virgin catheter status. The randomization schedule has been developed and will be administered by an independent trials unit. The pharmacy departments at each study site will receive the randomization number and treatment allocation via e-mail, and will prepare and dispense the drug accordingly. The study investigators, coordinators, patients, and all members of the research team will be blinded to treatment allocation.

On treatment days the pharmacy will dispense four syringes to the study patient, to maintain blinding. For patients on the treatment arm these syringes will contain rt-PA and saline (for the "top up"), numbered according to order of instillation. To ensure blinding, patients in the control arm will also be dispensed four syringes all of which will contain heparin 5,000 units per ml. The volume of heparin used as the locking solution will be equal to the luminal volume. All syringes will be labelled and numbered by the pharmacy as to the order of instillation, and will be identical in appearance. On non-treatment days, heparin (5,000 units per ml, luminal volume), will be prepared and administered by the hemodialysis nurse, as per usual standard of care.

### Primary outcome

The primary outcome is catheter malfunction, defined by one of the following events occurring after attempts to re-establish patency have been undertaken as per a pre-defined protocol (including flushing the catheter with a 10 cc syringe, repositioning the patient and reversal of lines):

• Peak blood flow ≤ 200 ml/min for 30 minutes during an ongoing dialysis treatment (with maximum arterial and venous pressure limits of -250 mmHg and +250 mmHg respectively)

• Mean blood flow ≤ 250 ml/min during two consecutive dialysis sessions, calculated as the blood processed in millilitres divided by the time on dialysis in minutes (with maximum arterial and venous pressure limits of -250 mmHg and +250 mmHg respectively)

• Inability to initiate dialysis with no blood flow

According to the K/DOQI guidelines an extracorporeal blood flow of ≥ 300 ml/min is required to provide adequate dialysis [7]. A peak blood flow of ≤ 200 ml/min for 30 minutes, or a mean blood flow of ≤ 250 ml/min during two consecutive dialysis sessions, were chosen to define catheter malfunction as this blood flow will not permit achievement of adequate dialysis. The blood flow must be maintained with a maximum venous pressure of +250 mmHg and arterial pressure of -250 mmHg, to ensure higher blood flows are not achieved at the expense of excessive pressures. A blood flow which cannot be maintained with venous and arterial pressures of 250 mmHg should be investigated for catheter malfunction.

Reversal of catheter lines may be required to initiate or maintain dialysis during some sessions, however reversal of lines over several sessions may lead to increased re-circulation of blood and inadequate dialysis. The need for ongoing reversal of catheter lines to maintain dialysis would therefore suggest potential malfunction. A protocol regarding catheter line reversal will be implemented to ensure that ongoing line reversal is not undertaken, specifically:

• Catheter lines may be reversed in an attempt to re-establish patency and initiate/maintain dialysis

• Catheter lines may be reversed for **no more than three consecutive** dialysis sessions

• After three consecutive dialysis sessions with reversed lines **the next two consecutive** sessions must be run with lines in the straight position. Primary outcomes will then be assessed

• Primary outcome criteria is the same during the period of line reversal

• A dialysis session is considered to have included line reversal if **any portion** of that run was undertaken with a reversal of lines

### Secondary outcome

Catheter-related bacteremia, the secondary outcome, will be defined according to the Canadian definitions for catheter-related infections [31], as outlined in Table 2. Both "definite" and "probable" infections will constitute a study outcome. These patients will remain in the study and their catheter-related infection will be treated by the attending staff nephrologist according to usual practice. If a new catheter is clinically indicated the patient will be censored at the time of catheter removal.

**Table 2:** Definitions for catheter-related bacteremia

***Definite *catheter-related bacteremia**:
• Confirmation of septic thrombophlebitis with a single positive blood culture, or
• Single positive blood culture and positive culture of catheter segment with identical organism, or
• 10-fold colony count difference in blood cultures drawn from catheter and peripheral blood, or
• Single positive blood culture and positive culture from discharge or aspirate from exit site, tunnel, or pocket, with identical organism
***Probable *catheter-related bacteremia**:

• Two or more positive blood cultures with no evidence for source other than catheter, or
• Single positive blood culture for *S. aureus *or *Candida *with no evidence for source other than catheter, or
• Single positive blood culture for *coagulase negative staphylococci, Bacillus, Corynebacterium jeikeium, Enterococcus, Trichophyton*, or *Malassezia *in immunocompromised or neutropenic host or in patients receiving TPN with no evidence for source other than catheter

### Data collection

A web-based electronic case report form (eCRF) for data entry and ongoing communication and documentation will be used. Baseline data and ongoing data collection, as outlined in Table 3, will be obtained.

**Table 3:** Baseline and ongoing data collection

***Baseline data*:**
• age, sex, target weight, duration on dialysis, cause of renal failure
• type of catheter, insertion site, indication for catheter, history of rt-PA use or catheter replacement for malfunction
• details regarding previous catheter use
• history of deep vein thrombosis/pulmonary embolism
• baseline hemoglobin, platelets, albumin, Kt/V, urea reduction ratio (URR) and INR
• medication use
• comorbidities as defined by the Charlson Co-morbidity Index
• blood processed in millilitres
• time on dialysis (minutes)
• mean venous and arterial pressures
• number of dialysis set-ups (lines and membranes)
• mean intradialytic heparin dose
• weight loss
***Monthly Laboratory Data***:

• haemoglobin, platelets, albumin
• delivered dose of dialysis (Kt/V and URR)

### Patient follow-up procedures

Patients will be followed from trial entry until study completion (six month study period). The study coordinator will review each patient and their hemodialysis records at two week intervals and record primary or secondary end-points. Patients who reach the primary outcome of catheter malfunction will also be followed for an additional one month period, or until either: 1) six consecutive successful hemodialysis sessions are achieved (defined as a mean blood flow of ≥ 300 ml/min at each run in the two week period), or 2) the catheter is removed. Extending the follow-up will enable documentation of the natural history of the catheter after an episode of malfunction, and will allow an assessment of the costs associated with maintaining patency for the economic analysis. A time period of six successful hemodialysis sessions was chosen as recurrent malfunction typically occurs within two weeks of the initial malfunction [13]. Study drug may be discontinued for reasons of patient safety, or as requested by the patient. In these instances, the rt-PA will be discontinued but patients will be followed for study outcomes for the full six month study period. Reasons for discontinuation of study drug are outlined in Table 4.

**Table 4:** Reasons for Discontinuing Patient Study Drug

• Started anticoagulation therapy
• Major hemorrhage, defined as bleeding resulting in a drop in hemoglobin greater than 20 g/L or bleeding requiring blood transfusion with other clinical evidence or suspicion of bleeding
• Undergone major surgery
• Diagnosis of intraspinal or intracranial neoplasm
• Patient decision to withdraw
• Active pericarditis
• Missed two or more consecutive weekly study treatment sessions

### Study withdrawal

Given that the primary and secondary outcomes require the presence of a catheter, follow up for these outcomes can only occur if the patient is alive, accessible for monitoring, and has a catheter in-situ. Therefore patients will be censored and withdrawn from follow-up at the following points in time: removal of the catheter (including accidental removals and exchange over a guidewire); a non-mechanical indication (such as catheter-related infection, transfer to peritoneal dialysis, kidney transplantation, maturation of permanent access, or withdrawal from therapy); transfer of patient to another city which is not a satellite unit of the original study centre; and death.

### Statistical analysis

Analysis of the primary and secondary outcomes will be based on the intention-to-treat principle. Individual primary outcomes, in addition to the composite outcomes, will be examined. The time from randomization until catheter malfunction will be analyzed and Kaplan-Meier estimates of the probability of event-free survival will be calculated for the control and treatment arms. Cox proportional hazards analysis will also be used to compare event-free survival in the rt-PA and heparin groups, controlling for clinical risk factors and including stratification variables. A secondary on-treatment analysis will also be undertaken. A planned subgroup analysis will be undertaken stratified by catheter luminal volume. This analysis will take into account the actual concentration of rt-PA the patient received as the luminal volume, and thus rt-PA concentration, may vary between patients.

The proportion of patients in each treatment arm achieving the secondary outcome, catheter-related bacteremia, will be determined, as will the number of infections per 1000 catheter-days. Differences in proportions will be assessed using the Fisher's exact test and the number of infections per 1000 catheter days using the exact binomial test. A secondary analysis to identify predictors of catheter malfunction, including variables such as intra-dialysis weight loss and catheter flow parameters, will also be conducted.

### Sample size considerations

Sample size calculations are based on dropout rates and time to needing rt-PA for catheter malfunction as observed at the University Health Network in Toronto, Canada (C. Lok personal communication), as well as reported by Little et al [13]. The following parameters were used for the sample size calculations: annual dropout rate of 75%; one year event rate in the heparin arm of 95% and 98%; effect sizes of 30%, 33.3%, 35% and 40%; power of 0.80; and an α-level of 0.05. Calculations were done using simulations with 500 samples per condition, and assuming the dropout rate was independent of the primary outcome. Simulations including virgin versus non-virgin catheter status had no appreciable impact on sample size estimates. Table 5 presents the number of patients required per arm to detect specified risk reductions, at failure rates of 95% and 98% respectively. Given these estimates a study size of 170 patients per arm was chosen.

**Table 5:** Number of patients per arm, based on effect size and failure rate

**Effect Size**	**Failure Rate 95%**	**Failure Rate 98%**
**30%**	235	195
**33.3%**	185	160
**35%**	160	140
**40%**	120	95

### Cost-effectiveness analysis

The primary objective of the cost-effectiveness analysis is to determine the incremental cost required to avoid placement of a new hemodialysis catheter, assuming that prophylactic rt-PA is an effective strategy in preventing catheter malfunction. The secondary objectives are to determine which locking solution, rt-PA versus heparin, results in lower overall catheter-related costs and to determine the cost per month to maintain patency in a catheter for patients randomized to rt-PA compared to those randomized to heparin, after controlling for baseline covariates.

The resources required to maintain the patency of a hemodialysis catheter will include the following: the drug (ie rt-PA or heparin); catheter-related hospitalizations (for catheter-related bacteremia, associated complications of infections such as endocarditis and osteomyelitis and other catheter-related indications such as bleeding following catheter insertion); outpatient catheter-related infections; and outpatient treatment of catheter malfunction (i.e, rt-PA use and radiology procedures). The resources required will be collected for patients at all sites. Given that the cost of these resources may differ across sites, the cost of each resource will be assessed in detail at one site (Calgary Health Region). Drug costs will be obtained from the local pharmacy and provincial drug benefit lists as applicable. The cost of all other resources will be determined using the cohort of patients receiving care at the Calgary Health Region, using previously validated methodology [32].

Next, using the perspective of a health care provider, an economic evaluation, using decision analytic modeling, will be performed to determine the impact of heparin and rt-PA on costs and clinical outcomes (i.e, need for a new hemodialysis catheter and patient months with a patent catheter) and the cost required to avoid a new hemodialysis catheter placement over a one-year time horizon. A Markov process will be used to model monthly transitions between the three possible clinical states, "functioning catheter", "dysfunctional catheter" and death. The model outputs will be avoidance of a new catheter placement, patient months with a patent catheter, cost for each of the treatment strategies, and the cost to avoid placement of a new catheter for the use of rt-PA compared with heparin.

As described, although the number and type of resources required to maintain patients with a functioning catheter may be similar across the approximately 14 centres involved in this study, the costs of these resources may differ. To increase the generalizability we will therefore undertake a sensitivity analysis where the costs for catheter care related to hospitalizations and treatment of catheter malfunction are more representative of individual provinces, as well as other countries. This will be undertaken by obtaining fee schedules from each of the provinces represented in the study, as well as details regarding costs of catheter care available in the published literature.

## Competing interests

EBT has received payment for consulting from Hoffmann-La Roche. MJB, NSD and LM have received honorarium from Hoffman-La Roche for speaker fees.

## Authors' contributions

NSD and BRH were responsible for identifying the research question and drafting the study protocol. All authors have contributed to the development of the protocol and study design, as members of the research team. NSD and BRH were responsible for drafting of this paper and all authors provided comments and have read and approved the final version.

## Pre-publication history

The pre-publication history for this paper can be accessed here:


